# Development of Lab-Scale Continuous Stirred-Tank Reactor as Flow Process Tool for Oxidation Reactions Using Molecular Oxygen

**DOI:** 10.1021/acs.oprd.3c00424

**Published:** 2024-05-08

**Authors:** Ursina Gnädinger, Dario Poier, Claudio Trombini, Michal Dabros, Roger Marti

**Affiliations:** † Institute of Chemical Technology, Haute École d’Ingénierie et d’Architecture Fribourg, HES-SO University of Applied Sciences and Arts Western Switzerland, 1700 Fribourg, Switzerland; ‡ Department of Chemistry “G. Ciamician”, Alma Mater Studiorum, University of Bologna, Via Selmi 2, 40126 Bologna, Italy

**Keywords:** continuous stirred-tank reactor (CSTR), flow chemistry, oxygen, synthesis, free-radical annulation

## Abstract

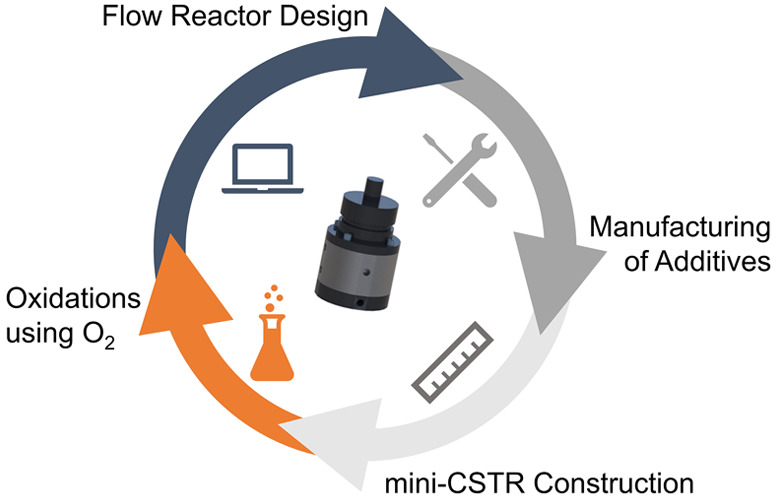

The use of sustainable oxidants is of great interest to the chemical industry, considering the importance of oxidation reactions for the manufacturing of chemicals and society’s growing awareness of its environmental impact. Molecular oxygen (O_2_), with an almost optimal atom efficiency in oxidation reactions, presents one of the most attractive alternatives to common reagents that are not only toxic in most cases but produce stoichiometric amounts of waste that must be treated. However, fire and explosion safety concerns, especially when used in combination with organic solvents, restrict its easy use. Here, we use state-of-the-art 3D printing and experimental feedback to develop a miniature continuous stirred-tank reactor (mini-CSTR) that enables efficient use of O_2_ as an oxidant in organic chemistry. Outstanding heat dissipation properties, achieved through integrated jacket cooling and a high surface-to-volume ratio, allow for a safe operation of the exothermic oxidation of 2-ethylhexanal, surpassing previously reported product selectivity. Moving well beyond the proof-of-concept stage, we characterize and illustrate the reactor’s potential in the gas–liquid–solid triphasic synthesis of an endoperoxide precursor of antileishmanial agents. The custom-designed magnetic overhead stirring unit provides improved stirring efficiency, facilitating the handling of suspensions and, in combination with the borosilicate gas dispersion plate, leading to an optimized gas–liquid interface. These results underscore the immense potential that lies within the use of mini-CSTR in sustainable chemistry.

## Introduction

The selective oxidation of organic molecules is a topic of crucial importance for chemical synthesis, both in academia and in industry.^1^ However, many of the classical protocols involve the use of stoichiometric amounts of toxic inorganic oxidants such as CrO_3_ and KMnO_4_, while more sustainable alternatives like Swern-type oxidation produce equivalent amounts of waste that have to be treated.^2^ Growing concerns regarding the resulting environmental impact led to pioneering advances in using molecular oxygen (O_2_) as the oxidant in synthesis,^3,4^ in the oxidation of organic molecules,^5,6^ and as a reagent either in its triplet ground state or singlet excited state.^7,8^ As the most atom-efficient and nontoxic reagent for catalyzed or direct aerobic oxidation reactions, it presents an incredibly sustainable alternative to other oxidants, e.g., the above-mentioned transition metal-based alternatives.^9^ Yet, safety concerns regarding fires and explosions when used in exothermic reactions, especially in combination with commonly employed organic solvents, limit its industrial use.^3,10,11^

With society’s growing ecological awareness and concerns about the industry’s environmental impact, it is of paramount importance to develop more sustainable chemical manufacturing protocols,^12^ i.e., utilizing renewable resources, and reducing waste and emissions. These require improvement of the chemistry and application of catalysis and the introduction of novel reactor technologies to aid the reaction performance and overall safety. An approach to tackle this challenge is a transfer from classical batch processing to continuous flow chemistry in milli- and microreactors.^13,14^ These designs offer the potential for more intensive process conditions and exploration of novel process windows^15^ owing to high mass and heat transfer rates originating from high surface-to-volume ratios.^16,17^ A variety of reactor concepts have been reported for gas–liquid biphasic systems, including tube-in-tube reactors,^18−20^ fixed bed reactors with static gas–liquid mixing,^21^ trickle bed reactors,^22^ thin film reactors,^23^ mesh microreactors,^24^ and spinning disk reactors.^25^ A consistent consideration with any system is to provide a sufficiently large gas–liquid interface through mixing to avoid low conversion rates, allowing the safe use of oxygen or air in flow.

Carrying out gas–liquid reactions in continuous stirred tank reactors (CSTRs) can overcome these limitations, as the CSTR offers the advantage of efficient mixing for gas−liquid reactions,^26,27^ as well as relatively simple construction.^28,29^ In particular, 3D printing, which has become a cutting-edge technology with enormous potential, proves successful in the fabrication of flow reactors. This rapid prototyping approach is effective in making custom reaction vessels for organic chemistry,^30,31^ especially in the case of flow chemistry.^32−35^ The unique properties of 3D printing technology for custom laboratory devices allow researchers to test and rapidly change the materials, geometry, and topography to adapt them to different chemical reactions. This results in an iterative approach where, in the development of greener reaction syntheses using molecular oxygen, the requirements of the chemical experiment determine the specific design and fabrication of the reactor device. The experimental results can be quickly incorporated into the reaction and reactor design and can lead to further modification or reactor optimization.

Here, we describe the development of a mini continuous stirred-tank reactor (mini-CSTR) enabling the safe and efficient use of O_2_ in organic synthesis. Residence time distribution (RTD) analysis demonstrates the ease of adjusting the reactants’ residence time through flow rate and module number. Investigation of gas–liquid mass transfer reveals the great stirring efficiency achieved through the magnetic overhead straining unit, even allowing for the operation with suspensions. Further, in combination with a high surface-to-volume ratio and integrated cooling, energy dissipation of exothermic reaction allows the safe use of O_2_ as an oxidant. Optimal O_2_ insertion is achieved by distribution over a borosilicate plate, installed in the bottom of the reactor. Taking advantage of state-of-the-art 3D printing technology to manufacture the mini-CSTR allows for free customization of the reactor design following the insights gained from experimental results.^3,36,37^ Two syntheses were tested to evaluate the reactor’s capabilities in flow chemistry: (i) the oxidation of 2-ethylhexanal to 2-ethylhexanoic acid using oxygen and (ii) the free-radical [2 + 2 + 2] cycloaddition of oxygen, an alkene, and ethyl acetoacetate to build an endoperoxide structure.

## Results and Discussion

### Mini-CSTR Design and Construction

**Figure 1:**
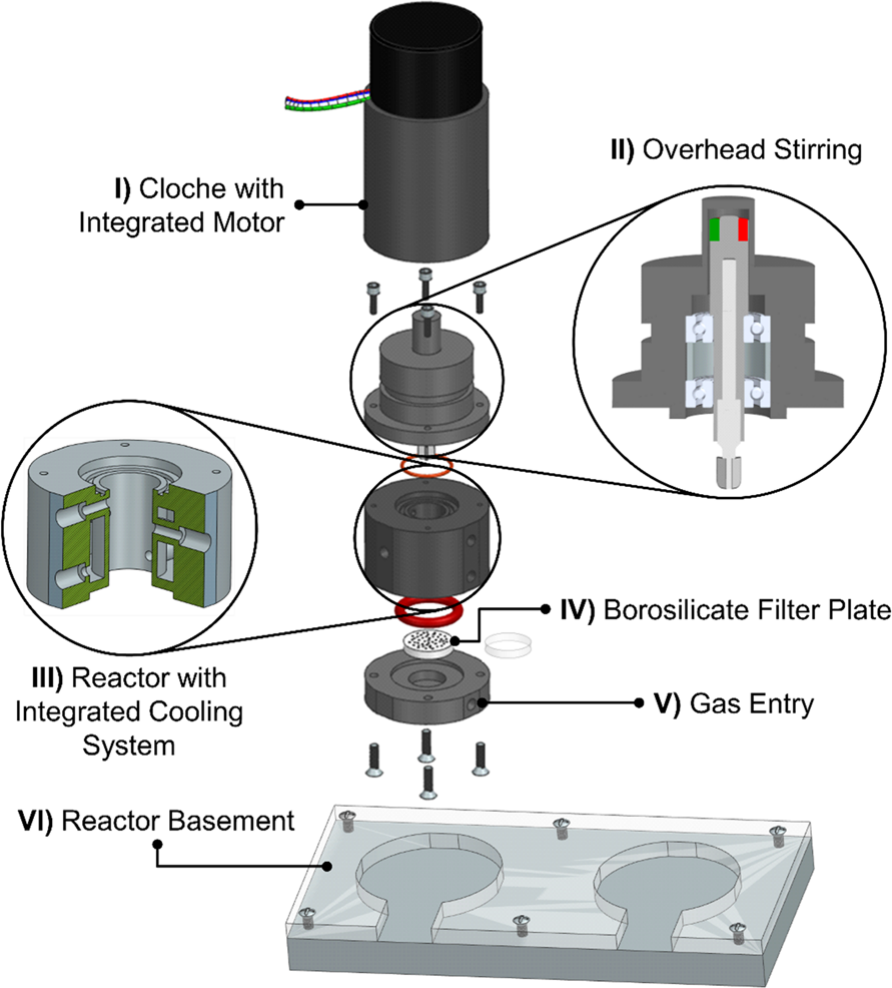
Exploded view of the designed mini-CSTR made by additive manufacturing. One module is composed of the following key elements: the overhead stirring unit via magnetic coupling (**I** and **II**), the 3D printed reactor middle part with integrated cooling (**III**), the gas dispersion with a borosilicate filter plate (**IV**) and gas entry unit (**V**), and the reactor basement (**VI**).

Inspired by Jensen’s work on mini-CSTR reactors,^36,38^ we built initial prototypes using various materials such as stainless steel or polysulfone and purchased the commercially available fReactor from Asynth.^26,39−41^ Tests in the laboratory on these reactors revealed some practical problems, such as inefficient mixing due to the use of magnetic stirring elements, especially for viscous or heterogeneous reaction mixtures as well as insufficient and difficult reaction temperature control. Based on this experience, our objective was to design and build a small double-jacketed CSTR unit with a better heating/cooling capacity and combine it with an overhead stirrer for intense active mixing. Practicality, simplicity, and versatility to adapt to different gas–liquid reactions have been established as important design criteria. To this effect, an efficient dispersion system for the gas phase was envisaged to be integrated into the design. Other important features of the newly developed flow process tool were its general chemical inertness, easy cleaning, small footprint, cost efficiency, and potential use in industrial applications. The basic component of each reactor unit consisted of three main parts (Figure 1). The lower part of the CSTR module contained the gas inlet and a borosilicate filter plate for efficient dispersion of the gas. The permeation of the gas phase occurred by pressuring the gas through the frit into the reactor chamber, which contains the liquid phase of the reaction. In this way, small bubbles were formed in the liquid, resulting in a larger specific interface surface and, thus, a good dissolving capacity. The frit is removable from the reactor module and, therefore, easy to clean or replace. To maximize the efficiency of the dispersion surface, the disc had the same diameter as the reactor so that the bubbles were formed over the entire reactor floor. Using an oxygen probe at the exit of the last reactor, sufficient oxygen abundance is asserted in the reactor modules, while a combination of a back-pressure valve and a high oxygen flow rate ensures high oxygen transfer into the liquid phase, as undissolved oxygen will be mixed into the solution in the subsequent units.

**Figure 2:**
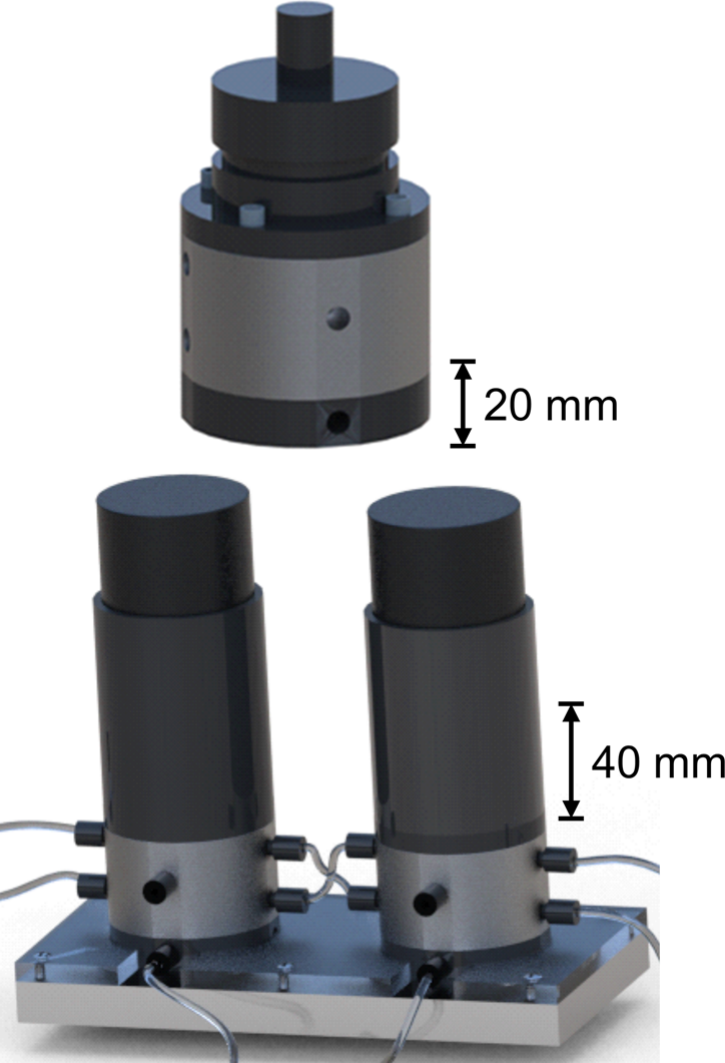
Sketch of the mini-CSTR setups. (a) The picture shows a mini-CSTR module. The 1/4–24 UNF threads for the gas feed, cooling liquid, and reaction mixture are visible. (b) Two mini-CSTR modules with the corresponding connecting tubes and the reactor platform.

The second key element is a cooling system integrated into the wall of the reactor unit (Figure 1). In the lid, a new design of a mixing system was developed, based on the concept of an overhead stirring system via magnetic coupling. This provided a hermetic separation between the product side and the environment, withstanding 5 bar of pressurized air at 100 °C without exhibiting any leakages or structural defects. When the motor rotated, the entire cloche adapter containing the two permanent cuboid magnets transmitted the torque via the cover to the magnetic ring connected to the stirrer. The mini-CSTRs and ancillary equipment were mobile and could be used alongside standard laboratory equipment (e.g., peristaltic pumps, back pressure regulators, and online or at-line monitoring; see Figure 2).

### Assessment of Mixing Properties by Residence Time Distribution (RTD)

Dead volume and bypass^42^ are two key factors that cause abnormalities in CSTRs and make it difficult to predict conversions for a given reaction. RTD measurements indicate such behavior and are important to know for a CSTR or a cascade of CSTRs. The measurements were performed under varying conditions, while gas insertion was deliberately omitted, providing a benchmark value for the mini-CSTR that facilitates comparison to other systems (see SI for experimental details). Since the injected tracer pulse, an Orange II solution, was not a perfect pulse, the outlet concentration profile was a convolution of the inlet concentration profile and the RTD.^36^ A model regression with the exponentially modified Gaussian distribution model (EMG)^43,44^ (equation S1) was used to extract the RTD function from the concentration profiles.

**Figure 3:**
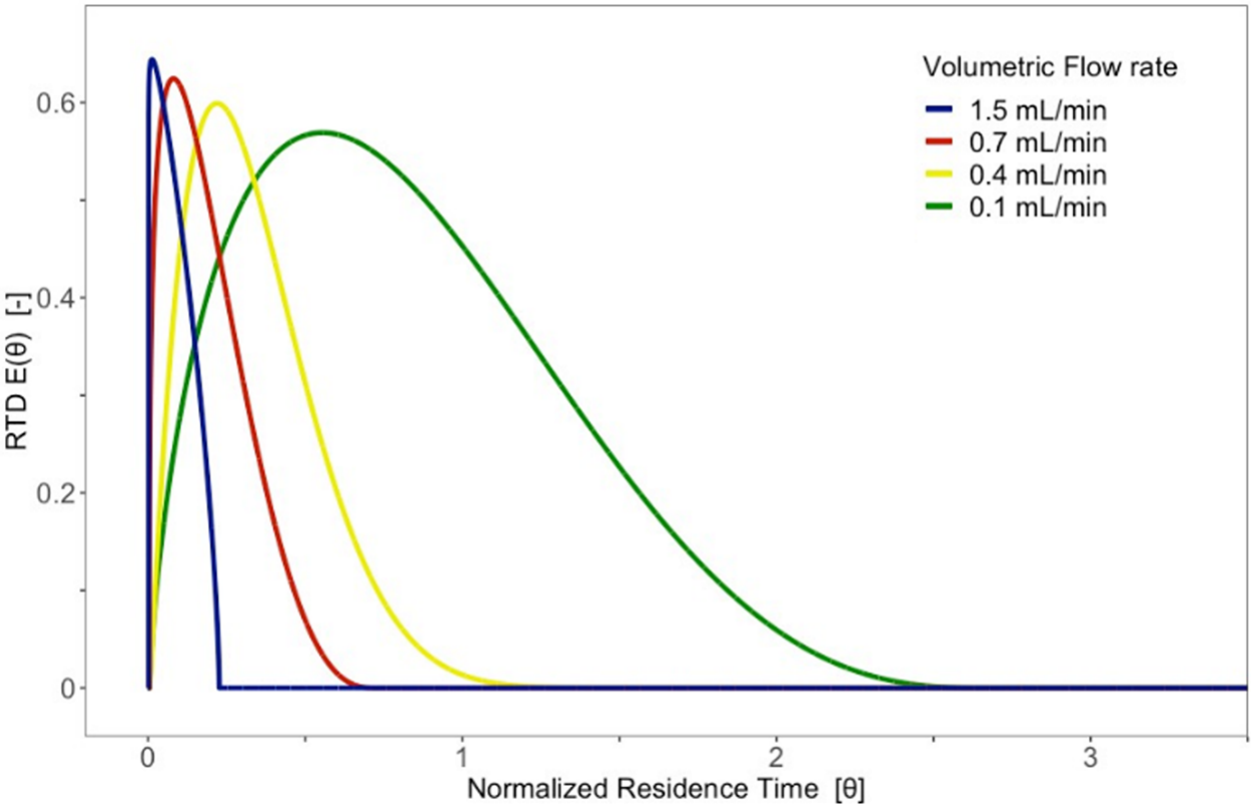
RTD *E*(Θ) function of the measurements 1.5, 0.7, 0.4, and 0.1 mL min^–1^.

The mixing performance of the mini-CSTR module was evaluated at varying flow rates. The average residence time was 228–2952 s at a liquid flow rate of 1.5 to 0.1 mL min^–1^ and a stirring speed of 800 rpm (Figure 3 and Table S3). In general, the determined residence times were lower than the theoretical values. A possible hypothesis is that the new mini-CSTR model contained a dead zone in which little or no material exchange took place. Chapman et al. reported results for their CSTR reactor that were closer to a normal distribution (theoretical RTD function).^26^ This could be explained by the smaller reactor volume (2 mL versus 6.14 mL) and the different design (no gas introduction via the sintering plate), similar to that of Jensen and Mo, who reported excellent agreement between experimental and theoretical RTD profiles for a cascade with up to seven CSTRs in series.^36^

As a result, the effectively used reactor volume (flow rate multiplied by the determined mean residence time), with an average value of 5.14 mL, was smaller than the nominal value of 6.52 mL. The calculation of the percentage filling confirmed this hypothesis, with 12.6–24.5% of the unused reactor volume (Table S3). Most likely, this is the volume of the mini-CSTR reactor located under the borosilicate filter plate. The calculated percentage of the total volume of about 21% confirmed the existence of a dead zone.^45^ However, this did not limit the reactor further, as the main application was to feed a gas component into the system, and thus no complete filling of the reactor was necessary.

### Determination of the Overall Heat Transfer Coefficient (UA)

A good and important indicator of heat exchange efficiency is the overall heat transfer coefficient UA^46^ of the double-jacketed reactor vessel. To determine this coefficient, a heat balance was established for one module resulting in a simplified UA (equation S2).

The overall heat transfer coefficient values were measured in the range of 244 to 536 WK^–1^ m^–2^, for temperatures of 20 to 25 °C, with flow rates of the reaction and cooling fluids ranging from 0.1 to 2.0 mL min^–1^ and from 5 to 18 mL min^–1^, respectively, and with stirring speeds of 400 to 1200 rpm (see the SI for detailed experimental protocols). This value of UA is lower than that of a plate heat exchanger but comparable or even better than a batch reactor of similar size.^28,47^

The following influence of the key parameters on the overall heat transfer coefficient was observed: reducing the cooling flow rate, reactor flow rate, and stirring speed led to an increase of the heat transfer resistance, which resulted in a reduction of the UA value. A general explanation for this phenomenon is the change in the temperature gradient across the reactor wall. In addition to the resistance created by the reactor wall, the heat transfer is influenced by the Prandtl boundary layer that forms over the inner reactor chamber wall.^48^ The more the flow rate or the stirring speed was reduced, the thicker the layer became and the resistance to heat transfer increased, which is reflected in a reduction of the UA values. An influence of the reaction temperature was not observed.

### Estimation of Gas–Liquid Mass Transfer Coefficient

Due to the low solubility of most gases, the rate-limiting step of the overall reaction resides in the gas–liquid mass transfer. In the newly developed mini-CSTR, oxygen was injected into the reactor. The gas was pressed through the borosilicate filter plate and fine bubbles built in the liquid phase, forming a gas–liquid contact surface. The stirrer promotes this gas–liquid contact by breaking up the rising gas bubbles and distributing them even further in the liquid volume. The constant collision and recirculation of the gas bubbles create a flow pattern of small bubbles that increase the contact area and time available for mass transfer, resulting in faster gas dissolution and eventually saturation. Knowledge of the gas–liquid mass transfer and reaction rates is crucial for achieving the desired yield and selectivity. The determination of the volumetric gas–liquid mass transfer coefficient^49,50^*k*_La_ is a good indicator of the gas–liquid mass transfer (equation S3).

**Figure 4:**
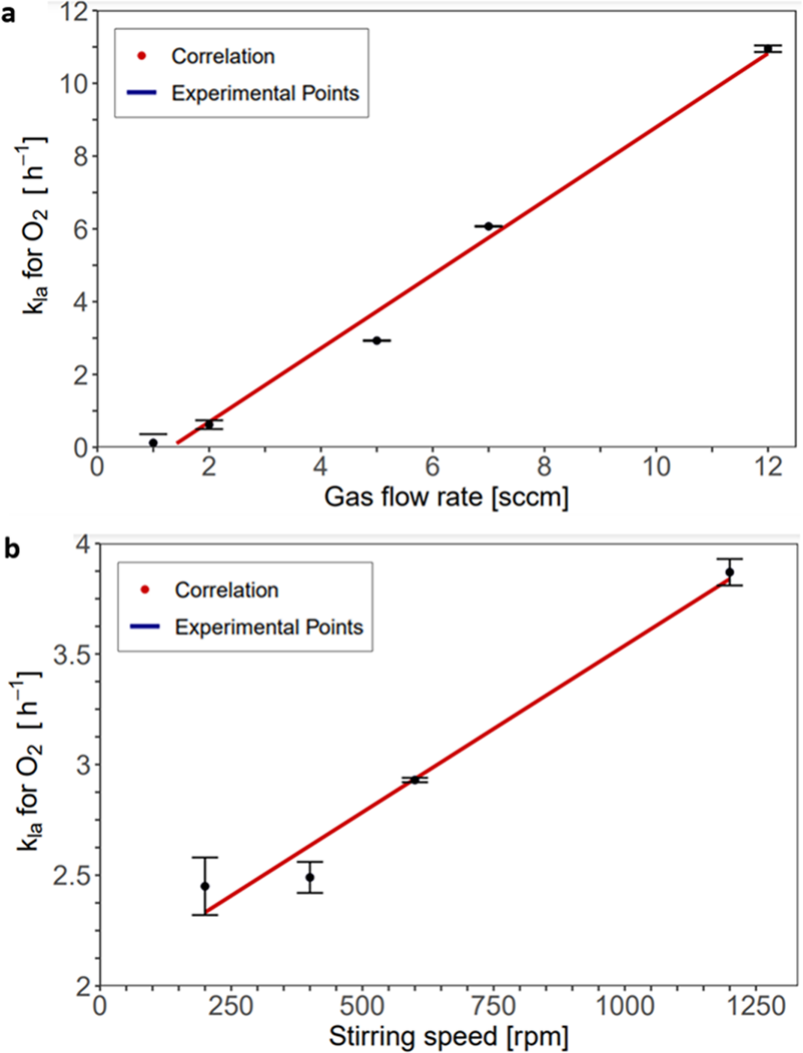
Determination of *k*_La_. (a) *k*_La_ as a function of gas flow rate with a stirring speed of 600 rpm and a liquid flow rate 0.7 mL min^–1^ (b) *k*_La_ as a function of agitation speed with a liquid flow rate of 0.7 mL min^–1^ and a gas flow rate of 5 sccm (at 20 °C and 1.01 bar).

The *k*_La_ value for O_2_ was determined in a range of 0.12–10.95 h^–1^ for a mini-CSTR module. One of the influencing parameters is mixing (Figure 4a) as the *k*_La_ for O_2_ increased from 2.49 to 3.87 h^–1^ when the stirring speed was increased from 400 to 1200 rpm. By increasing the gas flow rate (Figure 4b), the *k*_La_ value changed from 0.62 to 2.93 and 6.07 h^–1^ when the gas flow rate was increased from 2 to 5 and 7 sccm at a stirring speed of 600 rpm. At 12 sccm, the highest *k*_La_ value of 10.95 h^–1^ was reached. The higher oxygen supply leads to a higher amount of dissolved oxygen and thus to an improved reaction rate; similar results can be found in literature.^51,52^ Another analysis was carried out with variation of the reactor temperature. No significant differences were found between 15 and 25 °C. These values for *k*_La_ are lower than reported for a continuous agitated cell reactor (ACR) with 344 h^–1^, which can be explained because the ACR has 10 units connected in a series with a total volume of 100 mL.^53^

### Continuous Handling with Exothermic Conditions in the Oxidation of a Benchmark Aldehyde

**Scheme 1:**
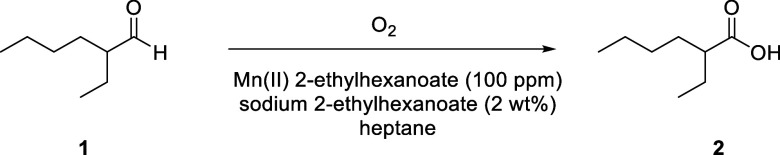
Oxidation of 2-Ethylhexanal (**1**) with Oxygen to 2-Ethlylhexanoic Acid (**2**)

**Figure 5:**
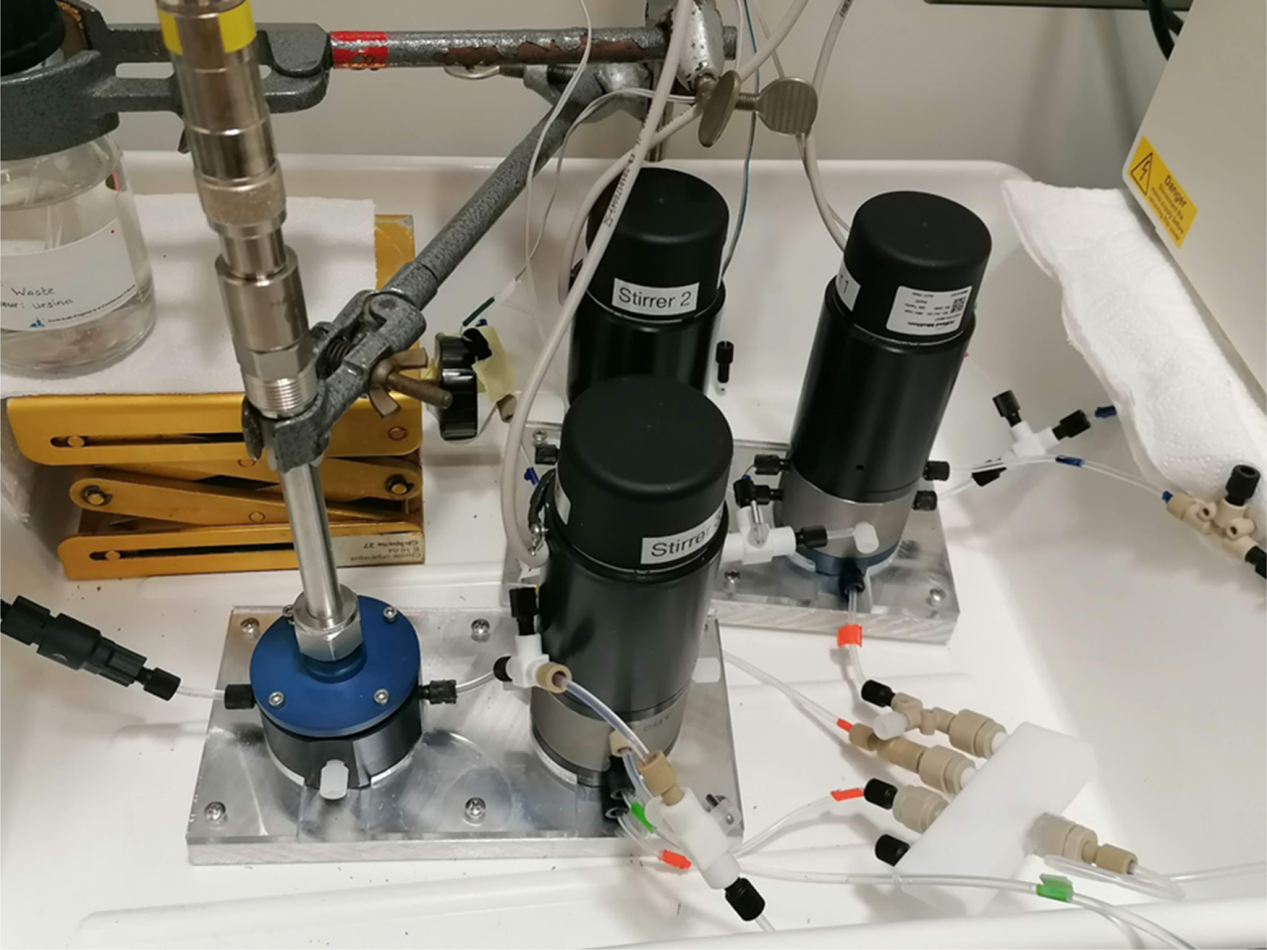
A picture of the complete setup consisting of three mini-CSTR modules and an oxygen sensor (blue lid, left side). The gas is introduced via 1/8″ tubing (marked in orange) in the lower part of the reactor after it has distributed through a splitter module.

The first reaction studied is the oxidation of 2-ethylhexanal (**1**) to 2-ethylhexanoic acid (**2**; Scheme 1). With a plethora of applications in catalyst, resin, stabilizer, pesticide, and emulsifier production, 2-ethylhexanoic acid (**2**) is produced through a multistep reaction on an industrial level, with the final step being the oxidation of the corresponding aldehyde. Within this application, the challenge lies in providing a reaction environment that is sufficiently saturated with oxygen to avoid performance decline originating from the system’s oxidant deficiency. A test of the mini-CSTR’s ability to handle an exothermic reaction uses up to three mini-CSTR modules, with Mn(II) acetate as the catalyst (Figure 5). The liquid and gas flows were adjusted to use an excess of oxygen relative to that of the aldehyde. A slight exotherm was observed during the reactions, but this could be efficiently cooled with the integrated cooling jacket of the reactor unit of the mini-CSTR.

Increasing the retention time by adding further mini-CSTR, resulted in higher conversion (Table 1). The conversion was improved from 69 to 73% by changing from one to two modules. The highest conversion of 90% was achieved with three modules and a system pressure of 1.38 bar. Another approach to increasing the retention time and thus the conversion without adding more modules could be achieved by reducing the flow rate. In other work, conversion of up to 100% was achieved with a plug flow reactor (PFR) at a residence time of 17.4 min and 5 bar O_2_ in the catalyzed condition.^54^ A reason for the greater reaction rate could be the higher system pressure in the PFR resulting in a greater abundance of O_2_ in the liquid phase.^55^ Meanwhile, the selectivity was almost constant at 97 ± 1%, indicating that if the conversion rate is high enough to avoid the aldehyde reacting with the formed peracid, it is independent of the pressure and residence time. This gain in selectivity is considerable since, for reactions in batch and PFR,^54,56^ the selectivity for the carboxylic acid was in the range of 70 to 85%.

**Table 1:** Mn(II)-Catalyzed Aerobic Oxidation of 2-Ethyl-hexanal (**1**)

entry	no. mini-CSTR units	residence time [min]	conversion^a^ [%]	selectivity^a^ [%]	yield^a^ [%]
1	1	4.5	69	97	67
2	2	9	73	97	71
3	3	13.4	90	96	86

a Determined by GC-MS. Reaction conditions: O_2_/RCHO molar ratio, >2.5; aldehyde, 1.5 M; *q*_v,liq_, 0.5 mL min^–1^; *q*_v,oxygen_, 4.8 sccm; mini-CSTR; back pressure, 20 psi; stirring speed, 200 rpm; jacket cooling temperature, 15 °C; cooling liquid flow rate, 10 mL min^–1^.

### Mn-Catalyzed Three-Component Synthesis of an Endoperoxide Precursor of Antileishmanial Agents^57^

**Scheme 2:**
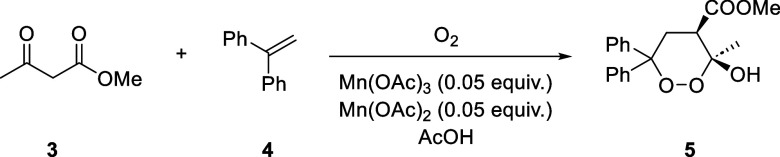
[2 + 2 + 2] Radical Cycloaddition Using Molecular Oxygen Forming an Endoperoxide

The Mn(III)/Mn(II)-catalyzed reaction of methyl acetoacetate (**3**), 1,1-diphenylethylene (**4**), and oxygen to form 3-hydroxy-3-methyl-6,6-diphenyl-1,2-dioxane-4-carboxylate methyl ester (**5**; Scheme 2) was carried out using one to three mini-CSTR modules.^58^ The reaction mixture was intrinsically more complex compared with the aldehyde oxidation. It consisted of three phases: a liquid phase (the acetic acid solution of the alkene and ketoester), a solid phase due to the partially soluble redox couple Mn(III)/Mn(II) in acetic acid, and the gas phase. The excellent diastereoselectivity observed in batch reactions was confirmed here by the formation of the cis isomer with a diastereoselective ratio (dr) > 9.1.

The liquid flow was regulated to maintain a constant Taylor flow, and excess oxygen was used relative to the reactants. The oxygen supply forced into the system through the filter plate results in a slightly turbulent medium, which prevents the sedimentation of solids. Another advantage proved to be the flexibility of using PFA tubing through 1/4–28 standard connections. The use of large tubing (1/8″), short connections between adjacent chambers, and fast stirring made transport without clogging possible over 3 h despite the presence of partially insoluble manganese salts and the formation of product particles. As the reaction progressed, the particle concentration in each chamber increased, leading to product particle growth along the flow direction, probably in the present reactor dead zones with low mass exchange. Future work will entail the continued improvement of the reactor design with a focus on minimizing low mass exchange sections, avoiding particle accumulation and, thus, system clogging during prolonged operation.

The conversion increased when additional CSTR modules were used as the total residence time increased. The increase from one to two reactor modules already led to an increase in conversion from 7 to 10% (Table 2). The highest result of 22% was achieved with three reactor modules with a residence time of 13 min. On the other hand, in a batch system there was a 90% conversion after at least 180 min reaction time. Work is in progress for process optimization using Design of Experiments to make use of mini-CSTR economical and competitive with batch technologies.

**Table 2:** Results and Experimental Conditions for Endoperoxide Synthesis^a^

entry	mode	*q*_v,liq_ [mL min^–1^]	*q*_v,g_ [sccm]^b^	residence time [min]	pressure [psi]	conversion^c^ [%]
1	batch			180	atm.	90
2	batch			180	atm.	85
3	mini-CSTR 1 module	0.5	4.8	4.5	20	7
4	mini-CSTR 2 modules	0.5	4.8	9	20	10
5	mini-CSTR 3 modules	0.5	4.8	13.4	20	22

a Reaction conditions: Taylor flow; stirring speed, 600 rpm; reaction temperature, 25 ± 2 °C; jacket cooling temperature, 20 °C; cooling liquid.

b Flow rate per module.

c Based on ^1^H NMR.

## Conclusion

A new, open-access, multistage continuous stirred tank reactor has been designed, constructed, characterized, and versatilely used in multiphase chemical processes. The mini-CSTR features three key elements: an integrated cooling system for efficient heat dissipation, a gas dispersion system using a borosilicate filter plate for diffusing gas into the liquid phase, and an overhead stirrer with a magnetic coupling system. Made of stainless steel, POM, and PTFE, its design offers flexibility, allowing for the easy cleaning and replacement of components. Its compact design enables easy adjustment of the reactor volume and number of units in a cascade, with universal HPLC fittings for compatibility with common lab equipment.

The homogeneous concentration and temperature profiles achieved by vigorous stirring in each chamber result in nearly ideal CSTRs with serial RTD profiles and accurate predictability of reaction conversions. To demonstrate the performance of our mini-CSTR setup, we performed a fast exothermic oxidation of 2-ethylhexanal (**1**) to 2-ethylhexanoic acid (**2**) with molecular oxygen with good conversion (90%) and extraordinary selectivity of 97% (compared to only 70–80% for batch reaction). This is mainly due to the fact that our mini-CSTR system can be operated with a very narrow RTD and a good oxygen distribution allowing for fast conversion in the presence of the manganese(II) catalyst, thus leaving only little unreacted aldehyde for the subsequent side reaction.

A preliminary study was also carried out in a much more complex reaction, the three-component cycloaddition of an alkene, a β-ketoester, and O_2_ to give a 1,2-dioxane **5** in acetic acid. Even though the reaction was much slower, despite the technological challenge of coping with a multiphase system, we obtained modest but encouraging results regarding conversion (22% at 13 min compared to 90% at 180 min in batch mode) but with the same good diastereoselectivity as in batch. With its efficient overhead stirring that prevents sedimentation and gas introduction under slight pressure resulting in a slightly turbulent reaction medium, our newly developed reactor has the potential to be a valuable future process tool. The highly flexible design makes it possible to obtain a system that can be used for different synthesis requirements, not only for reactions with oxygen but also for reactions with other gases. Mini-CSTRs are a viable tool for the laboratory and are well on their way to becoming an applicable technology for the effective continuous production of pharmaceuticals.

## Experimental Section

### Materials

All reagents and solvents used are commercially available from Sigma-Aldrich, Alpha Aesar, Acros Organics, abcr, Gute Chemie, Carl Roth, Thermo Fisher Scientific, and Brunschwig Chemie and were used without further purification. Nuclear magnetic resonance (NMR) spectra were recorded with a Bruker 300 Ultrashield spectrometer and referenced against the chemical shift of the residual protio-solvent peak (CDCl_3_: 7.26 ppm) for ^1^H NMR and the deuterated solvent peak (CDCl_3_: 77 ppm) for ^13^C NMR measurements. Tetramethylsilane (TMS) and 1,4-dimethoxy-benzene were used as internal standards. Infrared (IR) spectra were recorded on Bruker ALPHA. Melting and boiling points were determined with a BUCHI Melting Point M-560. Ultraviolet (UV) spectra were measured on a Thermo Fischer Scientific UV–vis Spectrometer Type Evolution 200. Gas chromatography–mass spectrometry (GC-MS) was performed on a Thermo Scientific GC 1300 coupled with a MS ISQ and a Mega-5 MS Plus capillary column (Crossbond, 30.0 m × 0.25 μm ID, 0.25 μm). Online monitoring of dissolved oxygen (DO) in the flow setup was done with two Hamilton optical dissolved oxygen sensors VisiFerm DO Arc with a polytetrafluoroethylene (PTFE) coated membrane cap (H2 cap).

A VaporTecSF-10 reagent pump was used to pump liquids; gas introduction was done via a Carbagas O_2_ pressure regulator (outlet pressure 0–15 bar) and an SHO-Rate “50” Brooks rotameter mass flow controller (0–150 mm; 150 mm = 4.312 L h^–1^ O_2_).

### Determination of Residence Time Distribution (RTD)

The RTD profiles of the mini-CSTR cascade under liquid phase conditions were obtained using the pulse injection method. The carrier phase was deionized (DI) water, and the tracer was Orange II. Offline UV–vis spectroscopy was used to determine the concentration profiles of the tracer at the reactor outlet. Different parameters, including stirring speed, flow rate, and temperature, were tested. The data analysis was performed with a custom-written R script.

### Heat Transfer Coefficient (UA)

The overall heat transfer coefficient was calculated with one mini-CSTR module. Tempered water was pumped through the module at a specific flow rate, and the same was done for the cooling liquid (ethylene glycol/water 4:6, v/v). The UA coefficient was determined using different flow rates of water pumped in the reactor and cooling liquid as well as different stirring speeds and reactor temperatures.

### Volumetric Gas–Liquid Mass Transfer Coefficient (*k*_LA_)

Degassed milli-Q water was pumped through the installation with a flow rate of 1.2 mL min^–1^ and a stirring speed of 400 rpm. The dissolved oxygen saturation (%DO) of entering water was controlled by the first oxygen sensor. Nitrogen was fed to the CSTR module using a borosilicate filter plate (porosity 1) at a flow rate of 20 sccm. When the %DO of the second sensor was less than 0.2%, the N_2_ flow was replaced by airflow. The step response and the aeration response experiments were conducted using different stirring speeds and air and liquid flow rates as well as different reactor temperatures. The %DO concentration was recorded at 3 s intervals until %DO saturation was reached. The calculations were performed with the help of a custom-written R script.

The reactor design including illustrations, manufacturing details and material properties, setup illustrations, and protocols for the reactor characterization as well as their results can be found in the Supporting Information (Figures S1–S4, Tables S1–S4).

### Oxidation of 2-Ethylhexanal (**1**) to 2-Ethylhexanoic Acid (**2**)

A back pressure regulator of 20 psi (1.4 bar) was used at the outlet. An additional reactor module was added at the end to perform an in-line %DO measurement using an oxygen probe. To perform the reaction, an aldehyde solution (1.5 M) was prepared by mixing 2-ethylhexanal (23.04 g, 0.18 mol), Mn(II) 2-ethylhexanoate (40% w/w, 36 μL, 100 ppm), and sodium 2-ethylhexanoate (2% w/w, 1.6 g) in *n*-heptane (91.6 mL) and stored under nitrogen. In the beginning, the reactor was purged with nitrogen and then flushed with heptane. The reaction mixture was injected at a flow rate of 0.5 mL min^–1^ at 24 °C, and the supplied O_2_ was supplied at a flow rate of 4.8 sccm/module. The modules were cooled with the cooling jacket while the stirring speed was set to 200 rpm. The postreaction stream was collected in a round-bottom flask, tested for peroxides using indicator strips,^59,60^ evaporated, and analyzed by GC-MS and NMR without any further purification.

### Free-Radical [2 + 2 + 2] Cycloaddition of Methyl Acetoacetate (**3**), 1,1-Diphenylethylene (**4**), and Molecular Oxygen

The reaction in flow was performed with the setup shown in Figure S8 by using up to three CSTR modules. A back pressure regulator of 20 psi (1.4 bar) was used at the outlet. An additional reactor module was added at the end to perform an in-line %DO measurement using an oxygen probe. To perform the reaction in flow, solution 1 consisting of 1,1-diphenylethylene (1.74 g, 10 mmol, 1 equiv) and methyl acetoacetate (2.15 g, 20 mmol, 2 equiv) in acetic acid (conc., 60 mL) and solution 2 consisting of Mn(OAc)_3_·2H_2_O (0.14 g, 0.5 mmol, 5 mol%) and Mn(OAc)_2_·4H_2_O (0.12 g, 0.5 mmol, 5 mol%) in acetic acid (conc., 60 mL) were prepared and stored under a N_2_ atmosphere. In the beginning, the reactor was purged with N_2_ and then flushed with acetic acid. Solution 1 and solution 2 were injected each at a flow rate of 0.25 mL min^–1^ at 24 °C, and O_2_ was supplied at a flow rate of 4.8 sccm per module. The modules were cooled with the cooling jacket (thermostat temperature 20 °C) while the stirring speed was set to 700 rpm. The postreaction stream was collected in a round-bottom flask and tested for peroxides using indicator strips.^59,60^ Afterward, the reaction mixture was carefully quenched with aqueous NaOH solution (10 M) until neutralization, followed by extraction with ethyl acetate (3 × 50 mL). The combined organic phase was washed with aqueous NaHCO_3_ solution (2 M, 3 × 100 mL), dried over Na_2_SO_4_, and concentrated on a rotary evaporator. The crude product was purified by column chromatography on silica gel (cyclohexane/ethyl acetate 8:2, v/v) to obtain 3-hydroxy-3-methyl-6,6-diphenyl-1,2-dioxane-4-carboxylate methyl ester (**5**) as a colorless solid. Spectral data are in accordance with the literature.^58^

Additional details comprising setup illustration and product characterization can be found in the Supporting Information (Figures S5–S10).

## Supporting Information Available

Details on materials and accessories for the mini-CSTRs, experimental procedures for the characterization of the mini-CSTR unit (mixing, heat transfer, gas–liquid transfer), and flow setup and conditions for the tested reactions including analytical data (PDF)

## Abbreviations

*t*: time (s)
UA: overall heat transfer coefficient [W K^–1^]
*ṁ*: mass flow rate [kg s^–1^]
cp: specific heat capacity [J kg^–1^ K^–1^]
*T*: temperature [K]
*RTD*: residence time distribution [−]
sccm: standard cubic centimeters
