# Supplementary material for: Development of Lab-Scale Continuous Stirred-Tank Reactor as Flow Process Tool for Oxidation Reactions Using Molecular Oxygen
Source: Org Process Res Dev. 2024 May 8;28(5):1860–8. doi: 10.1021/acs.oprd.3c00424 (PMC11110044; doi:10.1021/acs.oprd.3c00424)
Supplement: Supplementary file 1 — op3c00424_si_001.pdf [file op3c00424_si_001.pdf]

Supporting Information

Development of Lab-Scale Continuous Stirred-Tank Reactor as Flow Process Tool for Oxidation Reactions using Molecular Oxygen

*Ursina Gnädinger[a], Dario Poier[a], Claudio Trombini[b], Michal Dabros[a], Roger Marti[a]**

[a] Institute of Chemical Technology, Haute école d'ingénierie et d'architecture Fribourg, HES-SO University of Applied Sciences and Arts Western Switzerland, 1700 Fribourg, Switzerland.

[b] Alma Mater Studiorum, University of Bologna, Department of Chemistry "G. Ciamician", Via Selmi 2, 40126 Bologna, Italy

* Corresponding author

Roger Marti, E-mail: roger.marti@hefr.ch

Table of Contents

Abbreviations	2
Equations	3
Material for a mini-CSTR module	4
Compatible accessories to complete the mini-CSTR setup	5
Reactor Design	6
Procedure to assess the mini-CSTR mixing properties	7
Procedure to determine the mini-CSTR heat transfer efficiency	8
Procedure for gas-liquid mass transfer assessment of the mini-CSTR	9
General flow setup for performing chemical reactions	12
Procedure for the oxidation of 2-ethylhexanal to 2-ethylhexanoic acid	13
Procedure for the [2 + 2 + 2] cycloaddition of β-keto esters and 1,1-di-substituted alkene	15

Abbreviations

**General**	
API	Active pharmaceutical ingredient
CAS	Chemical abstracts service
CSTR	Continuous stirred-tank reactor
RTD	Residence time distribution
sccm	Standard cubic centimeters per minute (1 sccm = 1 mL min^{-1})
**Techniques**	
GC-FID	Gas chromatography flame ionization detection
GC-MS	Gas chromatography-mass spectrometry
NMR spectroscopy	Nuclear magnetic resonance spectroscopy
UV-Vis	Ultraviolet-visible spectroscopy
**Compounds & Polymers**	
AcOH	Acetic acid
DI water	Deionized water
EtOAc	Ethyl acetate
EtOH	Ethanol
MeCN	Acetonitrile
MePh	Toluene
Orange II	4-(2-Hydroxy-1-naphthylazo)benzenesulfonic acid sodium salt
PEEK	Polyether ether ketone
PFA	Perfluoroalkoxy alkane
POM	Polyoxymethylene
PTFE	Polytetrafluoroethylene

Equations

$$E(t) = \frac{\lambda}{2}\exp\left(\frac{\lambda}{2}(2\mu + \lambda\delta^2 - 2t)\right) erfc\left(\frac{\mu + \lambda\delta^2 - t}{\sqrt{2\sigma}}\right)$$

Equation S1. The equation describes a statistical model that combines two components: a Gaussian distribution for stochastic phenomena such as molecular diffusion and mixing, and an exponential decay distribution to account for delays, for example, due to mass transfer or reaction kinetics. In this context, μ represents the mean residence time, σ is the standard variance, δ is the standard deviation, and λ is the rate parameter of the exponential decay component. This type of function is often used to model an asymmetric distribution, particularly one with a broadening towards the rear (*React. Chem. Eng*. **2016**, *1*, 501-507).

$$UA = \dot{m}_c c_{p,c}(T_{c,IN} - T_{c,OUT}) \frac{ln\left(\frac{T_{h,OUT} - T_{c,OUT}}{T_{h,IN} - T_{c,IN}}\right)}{T_{h,OUT} - T_{c,OUT} - T_{h,IN} - T_{c,IN}}$$

Equation S2. In addition to the mass flow rate, $\dot{m}_c$, the corresponding temperatures are included, with $T_{h,IN/OUT}$, and $T_{c,IN/OUT}$ referring to the inlet (IN) and outlet (OUT) temperatures of the cold (c) and hot (h) fluids.

$$\ln\left(1 - \frac{C_L}{C_{O2}^*}\right) = -k_{La}t$$

Equation S3. The calculation of the k_{La} was done using equation 3 (see SI for experimental protocols). Here, C_{O2}^* and C_L are the saturation concentration of dissolved oxygen and the concentration of dissolved gas in the liquid phase, respectively.

Material for a mini-CSTR Modul

Table S1. Material used for construction of a mini-CSTR module.

mini-CSTR Module Set-up	Nr.	Product	Supplier
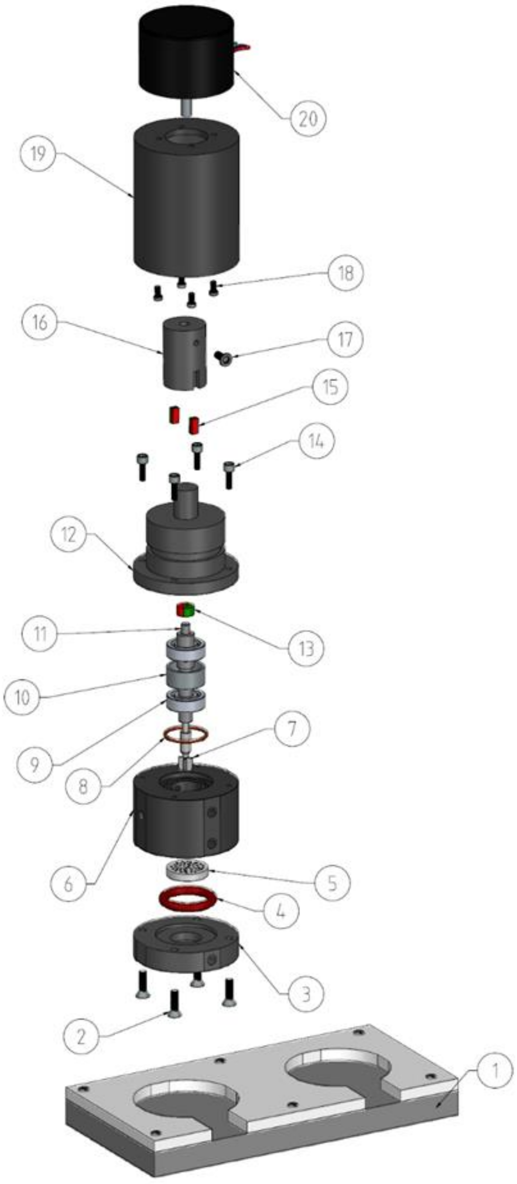	1	Reactor Basement	HEIA-FR
	2/18	Cylinder head screws to fix lids, M4×8, Inox A2, internal hex-socket cap-head	Distrelec
	3	Reactor bottom part, POM black	HEIA-FR
	4	Viton Seals, Ø 23.00×5.00 mm, 75 Shore	KUBO
	5	Filter plate with glass edge, Duran®, Ø 24 mm, height 4 mm, T_{max} = 450 °C, Porosity Nr. 2/ 3/ 4	Faust
	6	Reactor middle part, stainless steel 316 L	Ecopart
	7	Micro surface stirrer shafts, Ø 8 mm, length 120 mm, shaft dia. 3.5 mm, PTFE	Bola Chemical
	8	Viton Seals, Ø 21.95×1.78 mm, 75 Shore	KUBO
	9	Ceramic deep groove ball bearing 608-KER-LL-ZRO2-PEEK, size (inner×outer×width) 4×9×4 mm, ceramic zinc oxide	Sturm Präzision
	10	Reactor Ball Bearing Spacer Ring, POM black	HEIA-FR
	11	Reactor Stirrer Adapter, POM black	HEIA-FR
	12	Reactor Lid for magnet coupling, POM black	HEIA-FR
	13	Ring magnet R-10-05-05-DN, size (inner×outer×height)<br>5×10×5 mm, diamagnetic NdFeB	Supermagne te
	14	Cylinder head screws to fix lids, M3×12, Inox A2, internal hex-socket cap-head	Distrelec
	15	Quader magnet Q-08-04-03-N, size 8×4×3 mm, NdFeB	Supermagne te
	16	Reactor Cloche Adapter, POM black	HEIA-FR
	17	Cylinder head screws to fix lids, M5×16, Inox A2, internal hex-socket cap-head	Distrelec
	19	Reactor Cloche Adapter for Motor, POM black	HEIA-FR
	20	McLennan Servo Supplies Brushless DC motor up to 0.03 Nm, 24 V/ 12 W, Shaft-Ø 5.99×36 mm	RS Electronics

Compatible accessories to complete the mini-CSTR setup

Table S2. Accessories that are used in addition to the reactor in the flow setup.

Product	Supplier
Back Pressure Regulator Assembly 20 psi	Optimize Technologies
One-way Valve Inline Non-Metallic 1/4-28	IDEX
Flangeless ferrule ¼-28 flat-bottom for 1/16” OD Tubing, P200	IDEX
Flangeless ferrule ¼-28 flat-bottom for 1/8” OD Tubing, P300-x	IDEX
Flangeless fitting ¼-28 for 1/16” OD Tubing, XP-202	IDEX
Flangeless fitting ¼-28 for 1/8” OD Tubing, XP-301x	IDEX
Miniature 3-Way Stopcocks, PTFE, FEP, F731-04 – A, Connections 3 (female thread), for tubing I.D. × O.D. 1/16"×1/8"	BOLA
Miniature Distributors, PTFE, F710-11 – C, Connections 9, for tubing I.D. × O.D. 1/16"×1/8"	BOLA
PFA Tubing 1/16" OD × 0.10"	IDEX
Plug for ¼-28 flat-bottom Ports, P-309	IDEX
Pressure Regulator for O_2, outlet pressure range 0…15 bar	Carbagaz
Mass flow rate controller (MFC) for O_2, SHO-Rate “50”, regulation range 0…150 mm; 150 mm = 4.312 L h^{-1} O_2	Brooks rotameter
SF-10 reagent pump for liquid and gas	VapourTec
Thermostat Julabo FP50-Ma, range −10…100 °C, Bath volume 8 L, cooling solution: 40:60 Ethylene glycol:Water (*v*/*v*)	Julabo
Thermistor, NTC, 100 kΩ, BetaCurve-1 Series, Ø 1 mm, length 5 mm, T = 0…70 ± 0.2°C	Farnell
Tube Fittings T, PTFE, tubing I.D. × O.D. 1/16"×1/8"	BOLA
Optical dissolved O_2 sensor VisiFerm DO with PTFE coated membrane (H0 cap)	Hamilton

Reactor design

The reactor is a cylindrical block with a dimension of Ø 60 mm×100 mm (170 mm including the stirrer motor) with an inner diameter of the reactor 18 mm and a total volume of 6.52 mL. A total of five ports were drilled perpendicular to the reaction chamber to allow modular combinations of inlets and outlets. All connection ports have ¼-28 threads that can be attached directly to common IDEX fittings (IDEX Health & Science LLC.) without additional adapters, ideal for use with common laboratory equipment. All threads were hand-made after printing to achieve the desired precision. The stirrer motor can be mounted directly on the reactor top with a corresponding adapter. Since the bearings for the stirrer shaft and the impeller were located inside the container, compatible ceramic slide and roller bearings made of zirconium oxide were used. A commercially available mechanical cross PTFE-coated stirrer (Ø 8 mm) was used for mixing. The reactor middle section with the corresponding cooling system was 3D printed (ECOPARTS AG, Hinwil Switzerland) with stainless steel 316 L, having a non-insulating property and satisfying chemical stability. Polyoxymethylene (POM) was used for the reactor bottom and lid, as it is inexpensive and compatible with most solvents/reagents (except strong acids/bases). For constructing the reactor prototype, 3D sketches were created with the computer-aided design (CAD) of NX Siemens. The reactor base and the reactor lid with the overhead stirring system were manufactured by milling due to the simple geometry. Another major element is the gas dispersion system, achieved by installing a porous borosilicate filter plate on the reactor floor. By applying pressure, gas can be dispersed through the plate into the liquid phase. The flexibility of the design allows the user to remove and clean the plate or replace it with a filter of different porosity. By changing the porosity of the plate the bubble size and thus, the gas-liquid interlayer can be affected as well as the probability of pore plugging is reduced when using insoluble fine powders in the reactors, e.g., solid-supported catalysts.

At the time of writing (2023), the material costs for one module are currently amounting to 3’500 CHF:

- 3D printed reactor, made of 316L stainless steel 1’500 CHF
- overhead stirring unit with magnetic coupling 750 CHF
- Joints, stirrer, frits, fittings 750 CHF
- Electric control box for stirrer 500 CHF

Procedure to assess the mini-CSTR mixing properties

The measurement of the residence time distribution (RTD) was carried out using the pulse injection method and the setup shown in Figure S1. Deionized water was introduced as carrier liquid using a peristaltic pump and a 10-second pulse injection of Orange II (λ_{max} = 485 nm) was performed. The tracer was analyzed by offline UV-Vis spectrometry and mean residence time was interpolated using a custom R script. Different stirring speeds (200, 400, 800, and 1200 rpm) were tested at a volumetric rate of 700 μL min^{-1}, as well as different volumetric rates of 0.05, 0.1, 0.4, 0.7, 0.9, 1.2, and 2.0 mL min^{-1} at 800 rpm were also examined.

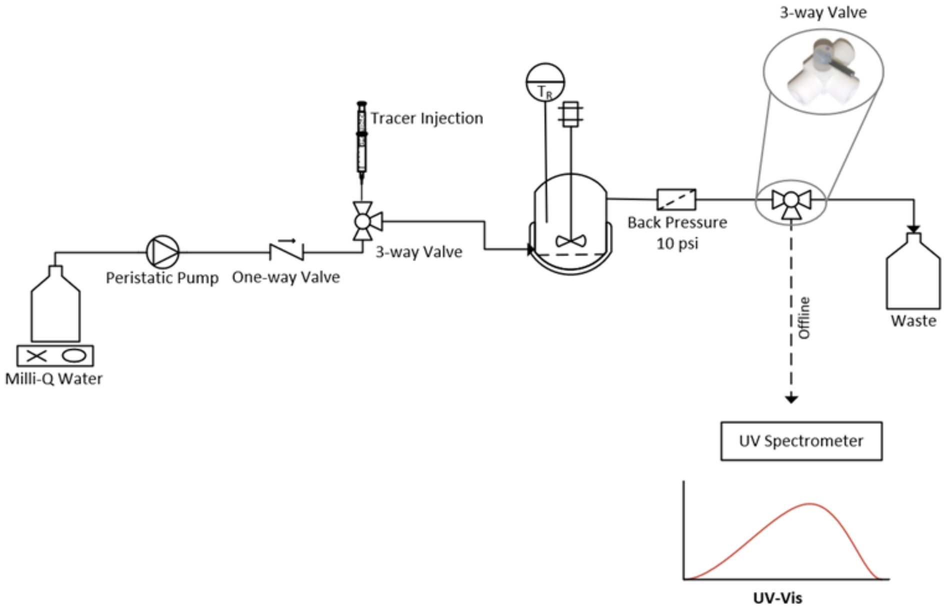

Figure S1. Set up for RTD Determination.

Table S3. Results of the residence time distribution investigation by varying flow rate $q_{v,liq}$.

$q_{v,liq}$ [ml min^{-1}][a]	t [s]	V_{used} [ml][b]	%filled[c]	τ_{ideal}[d] [s]	σ^2 [-]	s^3 [-]
0.1	2952	4.9	75.5	3912	1.40E+06	3.87E+04
0.4	783	5.2	80.1	978	2.38E+05	1.44E+04
0.7	485	5.7	86.8	559	1.05E+05	4.77E+03
1.5	228	5.7	87.4	261	2.11E+04	3.88E+02

[a] measured at a stirring speed of 800 rpm.
[b] calculated by $V_{used} = q_{v,liq} \cdot \tau$.
[c] calculated assuming a reactor volume of $V_{reactor}$ = 6.52 mL.
[d] calculated by $\tau_{ideal} = V_{reactor} \cdot q_{v,liq}$.

Procedure to determine the mini-CSTR heat transfer efficiency

The overall heat transfer coefficient (UA) determination was performed on one mini-CSTR module by introducing tempered water in the reaction chamber at a specific flow rate while doing the same for the jacket with ethylene glycol and water (4:6, *v*/*v*). The experimental setup is shown in Figure S2. The temperature IN/OUT of both the jacket and reaction chamber was recorded using Pt100 sensors in a T-module and a custom LabView script. The UA coefficient was determined at different volumetric rates (0.1, 0.4, 0.7, 0.9, 1.2 and, 2.0 ml min^{-1}) for the reaction chamber, different volumetric rates of the cooling liquid (5, 10 and, 18 ml min^{-1}), different stirring speeds (400, 800 and 1200 rpm) and different temperatures of the reaction chamber (20, 25, 30 and 35 °C).

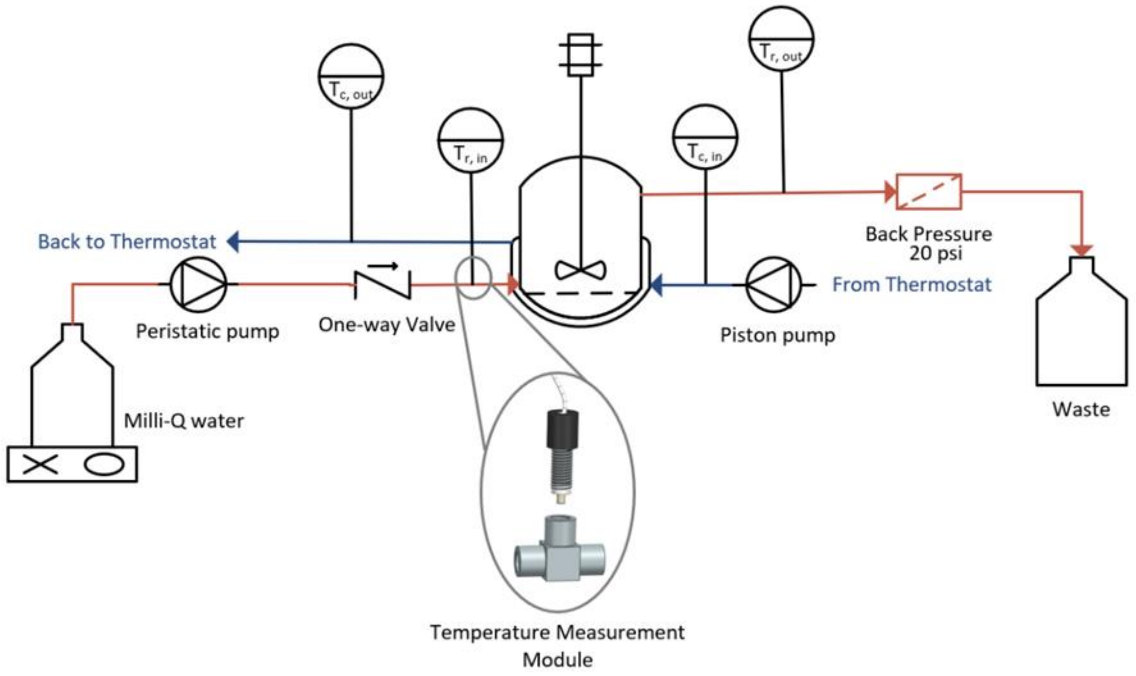

Figure S2. UA determination setup, consisting of one mini-CSTR module and four temperature sensors. Blue lines indicate the cooling liquid, while red lines indicate the reaction fluid.

Procedure for gas-liquid mass transfer assessment of the mini-CSTR

The determination of the gas-liquid volumetric mass transfer coefficient (k_{La}) was performed using the setup shown in Figure S3. Two Hamilton optical dissolved oxygen sensors VisiFerm DO with a PTFE-coated membrane were used (%DO Entry and %DO Exit). Before the experimental runs, a two-point calibration using air and nitrogen was carried out.

System equilibration. Degassed and deionized water was introduced in the reaction chamber with a volumetric rate of 1.2 mL min^{-1} and a stirring speed of 400 rpm. The first DO sensor measured the oxygen saturation (%DO) of the entering water, while the second measured DO in the exiting water. Through the frit of porosity 1, N_2 was introduced in the reaction chamber and the %DO was monitored until the %DO of the second sensor was equal to or less than 0.2%.

Determination of k_{La}. The experiment was performed using stirring speeds of 200, 400, 600, and 1200 rpm, airflow rates of 1, 3, 5, and 12 sccm, volumetric rates of 0.2, 0.7, and 1.2 mL min$^{-1,}$ and jacket temperatures of 15, 22 and 25 °C. The step response, the aeration response as well and the dissolved oxygen percentage were recorded with a time interval of 3 seconds until DO saturation was reached. The k_{La} was interpolated using a custom R script.

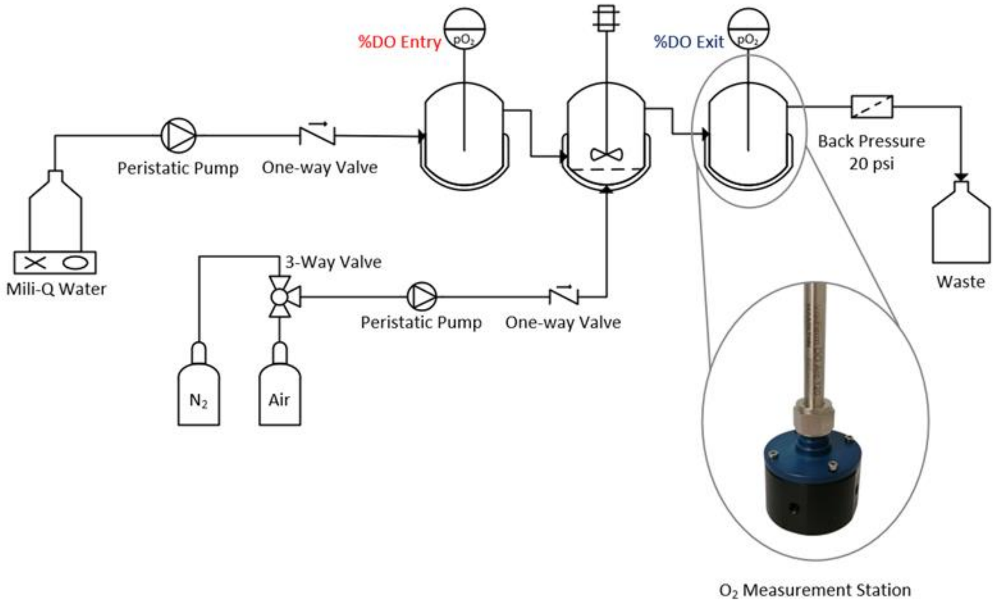

Figure S3. k_{La} determination setup.

a)

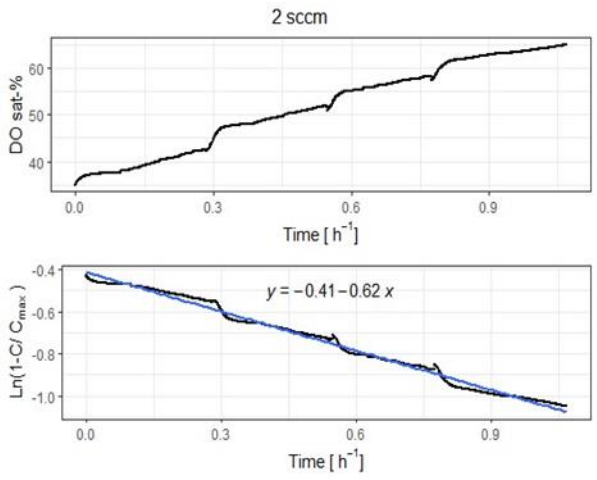

b)

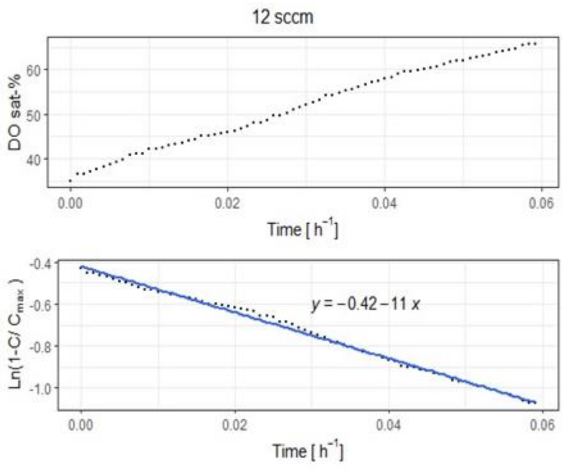

c)

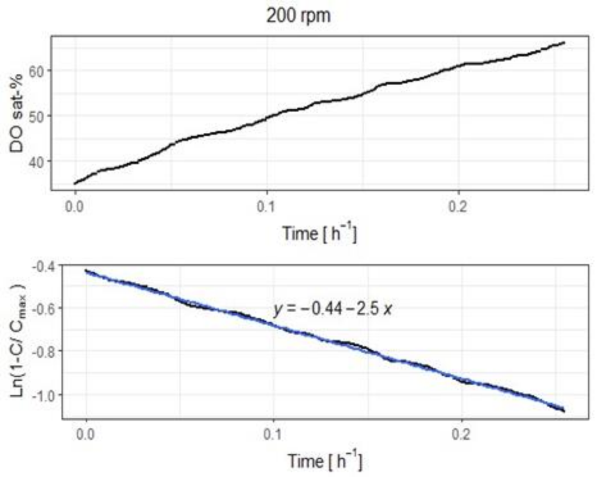

d)

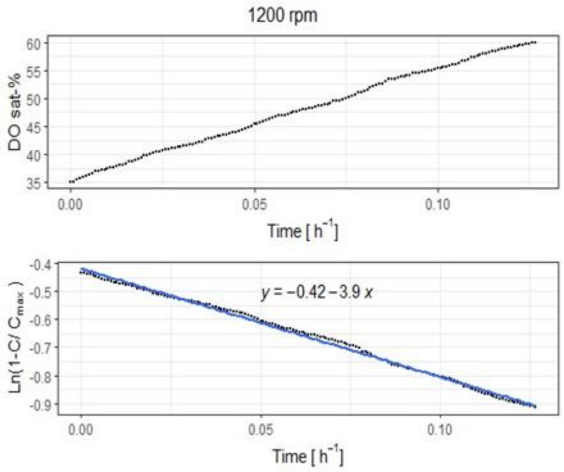

Figure S4. The linear measurement range of the measured data for the k_{La}: (a) 2 sccm; (b) 12 sccm; (c) 200 rpm; (d) 1200 rpm.

Table S4. k_La and τ for different gas flow rates $q_{v,g}$.

Entry[a]	$q_{v,g}$ [sccm][b]	k_La [h^{-1}]	τ [s][c]
1	1	0.12	9925
2	2	0.62	4836
3	5	2.93	1232
4	7	6.07	594
5	12	10.95	326

[a] stirring speed of 600 rpm, $q_{v,liq}$ of 0.7 ml min^{-1}, $q_{v,j}$ = 10 ml min^{-1}, $\vartheta_{r,IN}$ = 22 °C.
[b] at 20 °C and 1.01 bar.
[c] time at which 63% of the overall change in C_L is achieved.

Table S5. k_La and τ for different stirring speeds.

Entry[a]	Stirring speed [rpm]	k_La [h^{-1}]	τ [s][b]
1	200	2.45	1397
2	400	2.49	1489
3	600	2.53	1232
4	1200	3.87	915

[a] $q_{v,liq}$ = 0.7 ml min^{-1}, $q_{v,g}$ = 5 sccm, $\dot{\upsilon}_j$ = 10 ml min^{-1}, $\vartheta_{r,IN}$ = 22 °C.
[b] time at which 63% of the overall change in C_L is achieved.

General flow setup for performing chemical reactions

Main parts information for continuous flow setups:

1. O_2 gas cylinder 5 L, 99.9%, Carbagas
2. Pressure regulator for O_2, outlet pressure range 0…15 bar, Carbagas
3. Mass flow rate controller (MFC) for O_2, SHO-Rate "50" Brooks rotameter, regulation range 0…150 mm; 150 mm = 4.312 L h^{-1} O_2
4. SF-10 reagent pump of for liquid and gas, VapourTec
5. PFA tubings (OD 1/16 X ID 0.03'') with flangeless fitting ¼-28 and ferrule ¼-28
6. Thermostat Julabo FP50-Ma, range −10…100 °C, bath volume 8 L, cooling solution: 40:60 Ethylene glycol:Water (*v*/*v*)
7. Miniature distributors for random gas splitting, Bola
8. One-way valve inline non-metallic ¼-28
9. T-module with integrated temperature probe: Thermistor, NTC, range 0…70 °C (in-house made)
10. mini-CSTR module: middle part 3D printed in 316L stainless steel, bottom and top part in POM or PPS (in-house made)
11. Miniature 3-way stopcocks, connections 3 (female thread for ¼-28 fittings)
12. O_2 sensor in extra module: Hamilton optical dissolved O_2 sensor VisiFerm DO with PTFE coated membrane (H0 cap) (in-house construction)
13. Back pressure regulators, 10 or 20 psi, Optimize Technologies

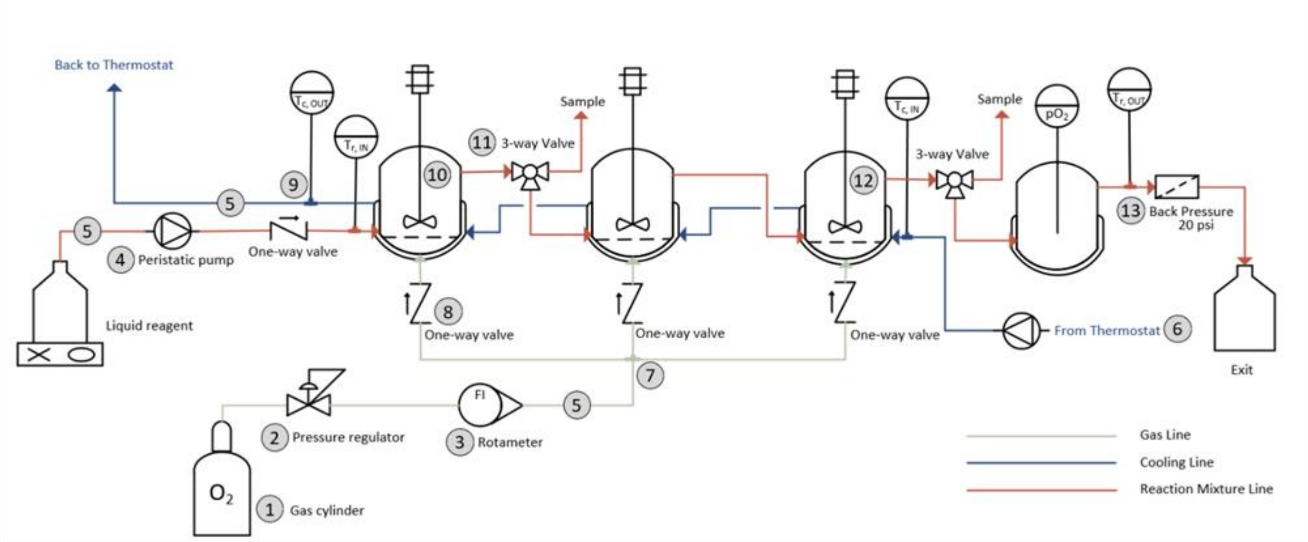

Figure S5. Flowchart of the general continuous-flow setup.

Procedure for oxidation of 2-ethylhexanal to 2-ethylhexanoic acid

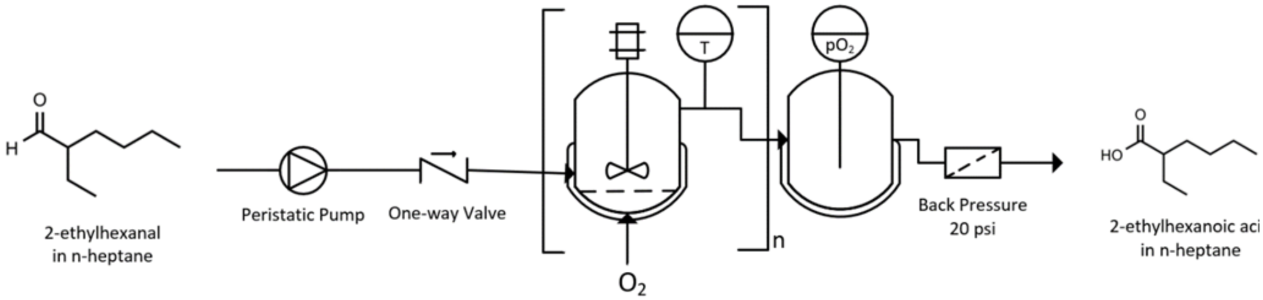

Figure S6. Schema of continuous-flow set-up for O_2-oxidation of 2-Ethylhexanal into 2-Ethyl-hexanoic Acid; Number of modules *n* is changing between 1 and 3.

GC-MS: The IPC method for the oxidation reaction was performed with an off-line GC-MS: Thermo Scientific GC 1300 coupled with MS ISQ, a Mega-5 MS Plus capillary column (Crossbond, 30.0 m×0.25 μm ID, 0.25 μm). The carrier flow was constant at 1.5 mL min^{-1} and the injector temperature was set to 350 °C. Temperature program: 3 min at 60 °C, then the temperature was increased by 3 °C min^{-1} to 150 °C and then by 20 °C min^{-1} up to 250 °C. Sample preparation: 400 μL sample and 50 μL internal standard solution 1000 ppm (dodecane) was diluted with 550 μL n-heptane. The conversion and selectivity to the carboxylic acid were determined based on the normalized peak areas for aldehyde, carboxylic acid, and by-products (Scheme S1).

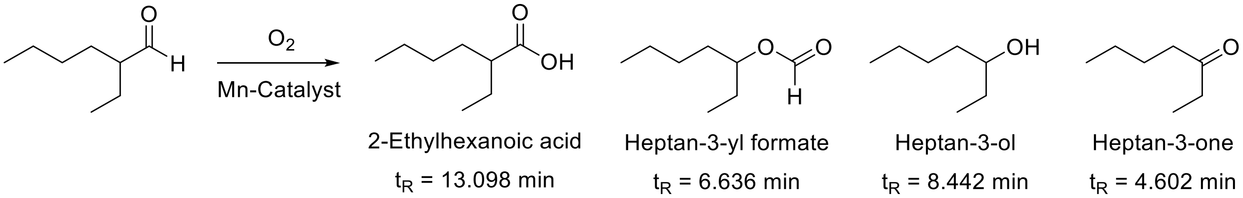

Scheme S1. Products formed in the oxidation of 2-ethylhexanal with GC retention times.

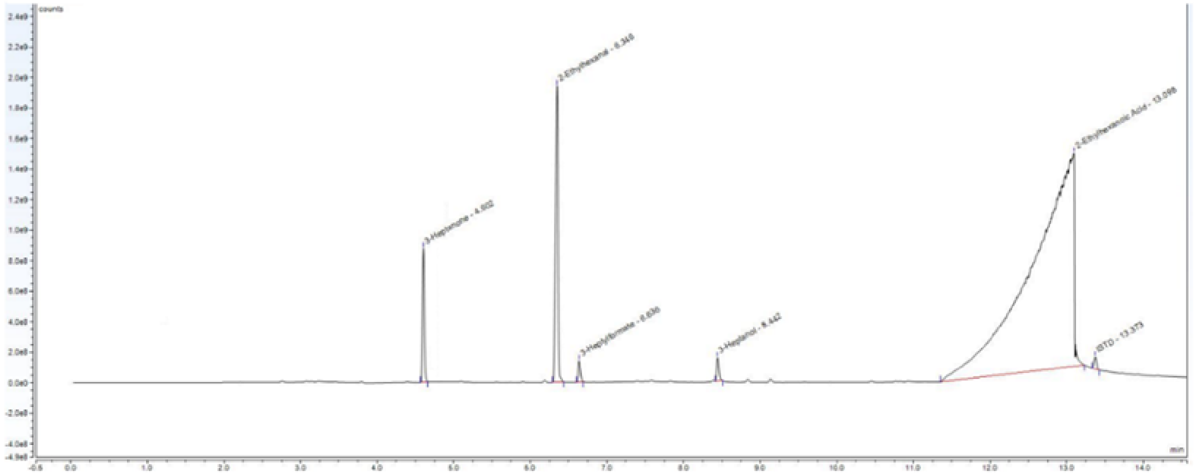

Figure S7. GC-MS Spectrum of a sample for O_2-oxidation of 2-Ethylhexanal.

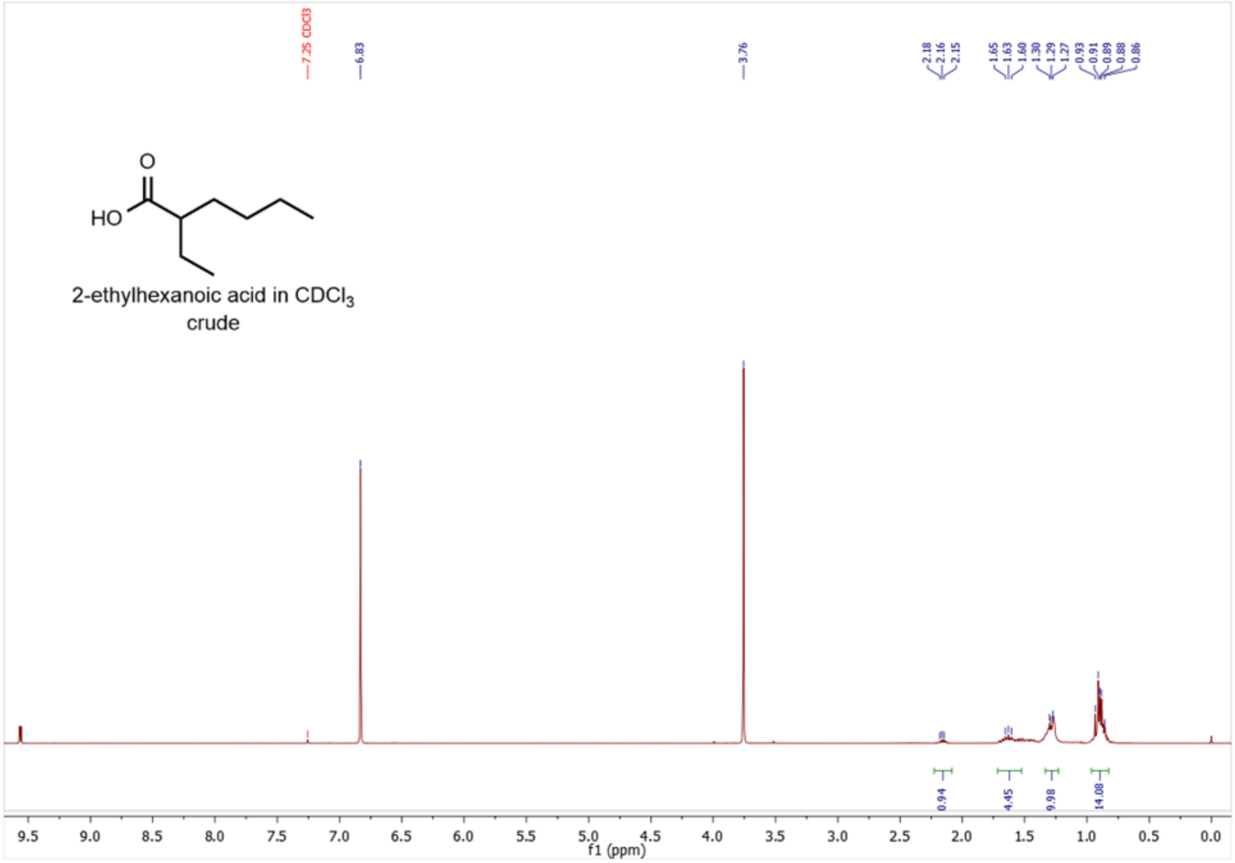

Figure S8. ^{1}H NMR Spectrum of a sample for O_2-oxidation of 2-ethylhexanal.

Procedure for the [2 + 2 + 2] cycloaddition of β-keto esters and 1,1-di-substituted alkene

For safe handling of endoperoxides careful DSC analysis is recommended to understand their thermal behavior (T_{onset}). Lardini et al. (*Org. Process Res. Dev*. **2021**, *25*, 2718–2729) use DSC analysis to show that an analog endoperoxide is stable at rt.

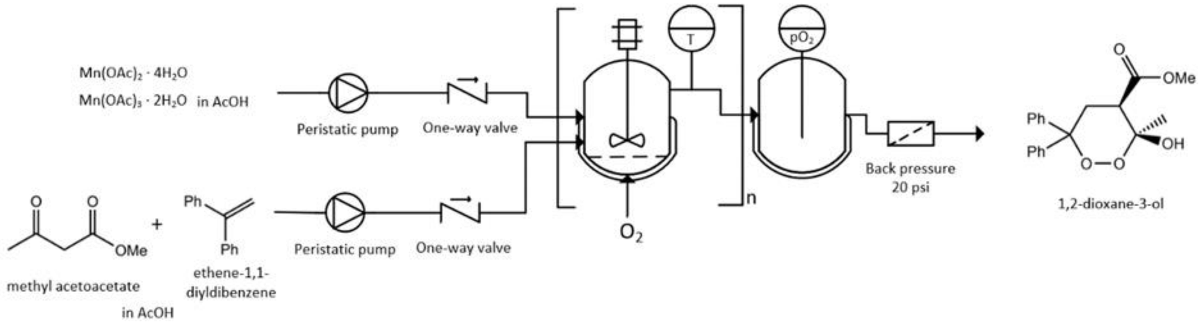

Figure S9. The continuous-flow set-up for endoperoxide synthesis. The number of modules *n* used in this study ranges between 1 and 3.

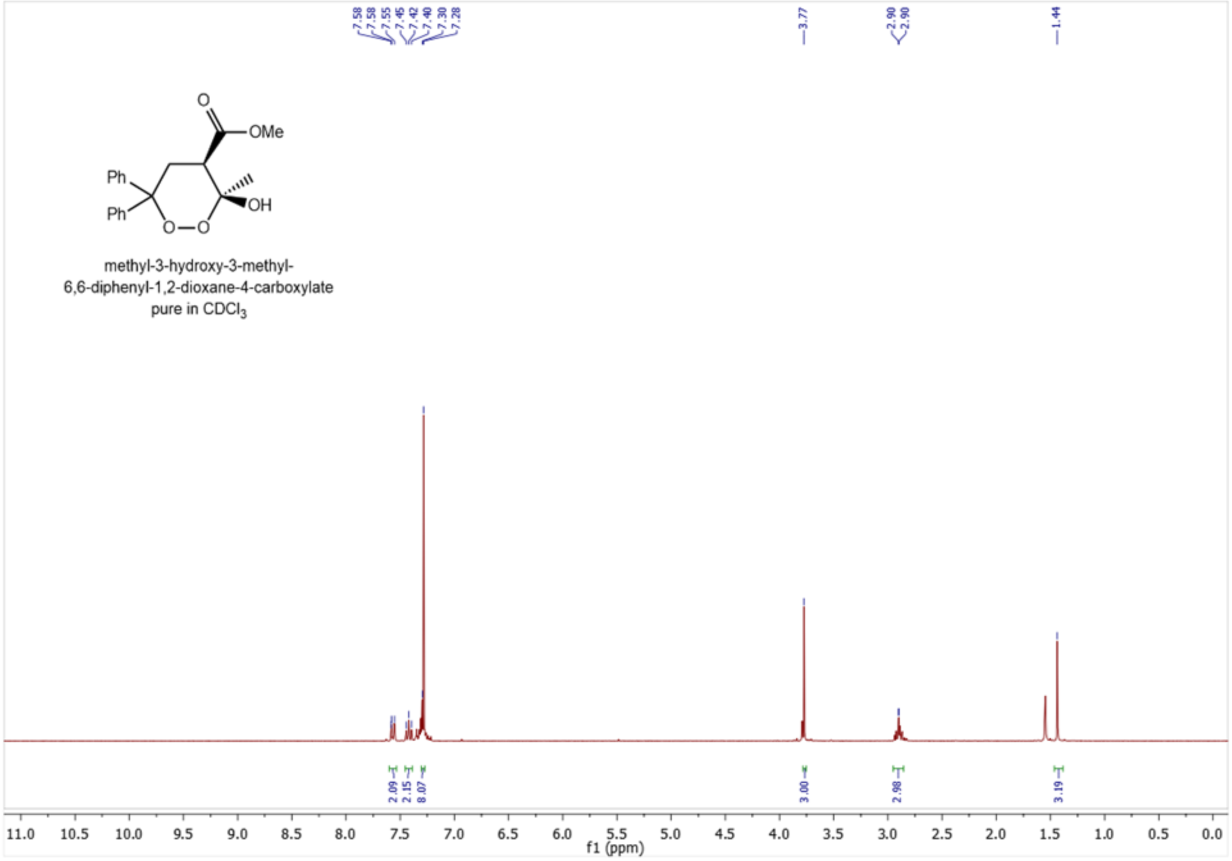

Figure S10. ^{1}H NMR Spectrum of a sample for endoperoxide synthesis.
